# Meso‐ and thermophilic posttreatment of press water coming from a thermophilic municipal solid waste digester

**DOI:** 10.1002/bit.28577

**Published:** 2023-10-30

**Authors:** Eva Maria Prem, Rudolf Markt, Mathias Wunderer, Andreas Otto Wagner

**Affiliations:** ^1^ Department of Microbiology Universität Innsbruck Innsbruck Austria

**Keywords:** amplicon sequencing, anaerobic digestion, biogas microbiology, temperature

## Abstract

An efficient biogas production out of organic (waste) materials is important to contribute to a carbon‐neutral future. In this study, thermophilic press water (PW) coming from an organic fraction of the municipal solid waste digester was further digested in a thermo‐ and mesophilic posttreatment approach using two semicontinuous 14 L digesters. The results showed that the PW can still have considerable high biogas potential—at least during the touristic high season in central Europe. The change in temperature led to an increase in volatile fatty acid concentrations and a decrease in biogas production in the mesophilic approach in the first days. However, the losses in biogas production at the beginning could be compensated thus there were no considerable differences in biogas production between thermo‐ and mesophilic posttreatment at the end of incubation. This can most probably be contributed to a change in the microbial community, and potentially problematic intermediates like valerate could be better degraded in the mesophilic reactor. Especially the abundance of representatives of the phylum *Bacteroidota*, like *Fermentimonas* spp., increased during mesophilic anaerobic digestion.

## Graphical abstract

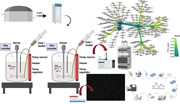

## 1 INTRODUCTION

Anaerobic digestion (AD) of biodegradable organic matter produces renewable energy in the form of biogas which can directly be combusted to gain heat and electricity, or upgraded to biomethane for natural gas grids and self‐ignition vehicles (Abanades et al., 2022; Gupta et al., 2023; Panwar et al., 2011; Plugge, 2017). In regard to the energy and climate crisis, biogas and biomethane are considered additional puzzle pieces in phasing out fossil fuels, and their contributions to the renewable energy sector are steadily increasing in Europe (Scarlat et al., 2018). According to the European Biogas Association (EBA), biogas/biomethane plants in Europe will double their productivity by 2030 and quadruple it by 2050 (EBA, 2020). The use of organic waste products like slaughterhouse by‐products (Moukazis et al., 2018) or agroindustrial residues (Pan et al., 2021) for AD is especially desirable as this approach is an effective, biocircular waste treatment, and thus an ethically and ecologically preferable procedure (Brémond et al., 2021; Dey et al., 2022; Lijó et al., 2017; Wagner et al., 2018).

The AD process is microbiology‐driven and consists of four functional steps: hydrolysis, acidogenesis, acetogenesis, and methanogenesis. Many different groups of microorganisms are involved in the AD process, and the sensitive balance among different groups of microorganisms is important for a stable and efficient AD process (Venkiteshwaran et al., 2015). The temperature regime can have crucial effects on physical, chemical, and microbiological parameters (Ahmed et al., 2021; De Vrieze et al., 2015; De Vrieze & Verstraete, 2016). To concurrently ensure process stability and high biogas yields, biogas plants are mostly operated at mesophilic (20–45°C) or thermophilic temperatures (45–60°C) (Chapleur et al., 2016), whereby mesophilic AD is the most common process (Kirchmeyr et al., 2021). In mesophilic AD plants, microbial communities are usually more diverse and more stable, intermediate toxicity is decreased, less energy is needed for heating, and propionate and free ammonia concentrations are lower than in thermophilic reactors. Conversely, the latter are characterized by higher diffusion rates, substrate solubility, hydrolysis rates, and volatile solids (VS) removal as well as shorter hydraulic retention times (HRTs); moreover, there is a higher feasibility of reactions at very low Gibbs' free energy changes, less phage infections, and decreased persistence of pathogens compared with mesophilic reactors (Ahmed et al., 2021; Dev et al., 2019; Gebreeyessus & Jenicek, 2016; Hejnfelt & Angelidaki, 2009; Kim et al., 2002; Labatut et al., 2014; Maus et al., 2016; Moset et al., 2015; Prem et al., 2020; Rosenwinkel et al., 2015; Shi et al., 2018; Speece et al., 2006; Suhartini et al., 2014; Wagner et al., 2008, 2009; Zeldes et al., 2015). A compromise was also proposed between these two temperature ranges (operation temperature: 45°C) to combine some advantages of both, meso‐ and thermophilic AD (Hupfauf et al., 2018, 2020).

There are only a few studies on the consequences of temperature changes during AD (Chapleur et al., 2016; Pap et al., 2015; Sudiartha et al., 2022; Sun et al., 2015). A temperature switch from mesophilic to thermophilic conditions resulted in significant differences in the microbial community, and genera like *Coprothermobacter*, *Bacillus*, *Caldicoprobacter*, *Methanobacterium*, and *Methanothermobacter* increased their abundance (Sun et al., 2015). However, in another study, a step‐wise increase in temperature from 42°C to 48°C showed that the microbial diversity only marginally changed despite the considerable decrease in biogas production at higher temperatures (Sudiartha et al., 2022). Chapleur et al. (2016) showed that an increase in temperature from 35°C to 55°C led to an increase in biogas production, whereas a decrease from 55°C to 35°C had negative effects on the overall biogas production even when the original inoculum derived from a mesophilic biogas reactor. Archaeal communities were more susceptible to temperature changes than bacterial ones, whereby the class *Clostridia* was exceptionally tolerant to temperature changes. *Methanosarcina* spp. (mesophilic), and *Methanothermobacter* spp. and *Methanoculleus* (thermophilic) were the most dominant methanogens (Chapleur et al., 2016). A full‐scale experiment was conducted with two 5430 m^3^ digesters which digested sewage sludge at 34°C and 50°C, respectively. After an adaption phase with reduced substrate input, the thermophilic digester produced more biogas and more nutrients could be released (De Vrieze & Smet, Klok, et al., 2016). When digesting food waste via single‐stage meso‐ and thermophilic as well as via temperature‐phased (55°C → 38°C) anaerobic digestion (TPAD), methane yields in the TPAD reactor were lowest; the authors attributed this to an insufficient sludge retention time. However, the energy conversion efficiency of the digested residue was higher in the TPAD than in the single‐stage mesophilic digester (Xiao et al., 2018). In another study using waste‐activated sludge, biogas yields and hydrolysis rates were higher in the TPAD (55°C → 35°C) than in the mesophilic reactor (Wu, Qin, et al., 2015). When digesting oily food waste, mesophilic and TPAD (55°C → 35°C, with or without recycle) reactors reached nearly identical methane yields (Wu, Kobayashi, et al., 2015). In conclusion, these studies showed that there are no simple conclusions regarding the effects of temperature changes during AD and that many factors (e.g., substrate, retention time) must be considered.

After biogas production of the organic fraction of the municipal solid waste (OFMSW), the digestate is often dewatered for composting to yield a valuable soil additive or is used for pyrolysis to produce biochar, bio‐oil, and syngas. The liquid fraction of the digestate contains valuable nutrients like nitrogen or phosphorous compounds and is thus further exploited via, for example, irrigation or hydroponics. To meet national requirements for further exploitation of the liquid fraction, mostly aerobic posttreatment procedures like composting, filtering, reverse osmosis, algae treatment, or biodrying have been applied to remove or transform undesirable compounds, fixate nitrogen, and reduce greenhouse gas emissions (Angouria‐Tsorochidou et al., 2022; Askri et al., 2016; Lu & Xu, 2021; Sfetsas et al., 2022). Another approach is to dewater municipal solid waste (MSW) of high moisture content (>60%), whereby the liquid fraction is anaerobically digested and solids are composted (Nayono, Gallert, et al., 2010; Nayono, Winter, et al., 2010).

Due to seasonal changes and thus fluctuations in substrate inputs during the year (Wagner et al., 2014), we hypothesized that liquid digestate (from thereon referred to as press water, PW) coming from a thermophilic OFMSW reactor in Roppen, Austria, could still have a sufficient methane production potential after thermophilic AD when substrate loads are high. To our best knowledge, the *biogas production potential of PW* coming from a thermophilic OFMSW digester has not been evaluated so far *by a second AD round* in general and under lower (thermophilic → mesophilic) temperatures in particular. The aim of this study was not to evaluate TPAD thus the separation of hydrolysis/acidogenesis (thermophilic) from acetogenesis/methanogenesis (mesophilic), but rather to establish a potential mesophilic, anaerobic posttreatment of PW during touristic high season. With the idea to save energy for heating and concurrently exploit the thermal energy of thermophilic PW in future anaerobic posttreatment approaches, we established two semicontinuously operated biogas reactors under thermophilic conditions and switched the temperature to 38°C in one of those reactors after adaption to lab conditions. In this context, we assessed the biogas yield of thermophilic PW at thermo‐ and mesophilic temperatures, and also evaluated other biochemical parameters as well as the composition and activities of microbial communities during thermo‐ and mesophilic AD.

## 2 MATERIALS AND METHODS

### 2.1 Reactor setup and experimental design

Two reactors with a respective working volume of 14 L were established. PW, used as inoculum and substrate, came from an OFMSW treating, thermophilic biogas plant in Roppen and was sampled in winter season 2017/2018 (Wagner et al., 2014). After sampling, the PW was immediately stored at 4°C. For better handling of PW during feeding and AD in the reactor, PW was 1:2 diluted with deionized water to prepare the initial reactor matrix. The airtight sealed reactors (*n* = 2) were constantly stirred at 170 rpm. The temperature was adjusted and controlled via a temperature sensor and a heating device laterally fixed on the respective reactor. For each reactor, the amount of produced biogas was measured with a standalone gas flow meter (BPC® µFlow). Each reactor was supplied with a sealable gas sack to avoid pressure fluctuations during sampling and feeding. On a weekly basis, the substrate was prepared by diluting reactor sludge 1:2 with deionized water and subsequently stored at 4°C. Prior to feeding, gas samples were taken and analyzed. Daily, 600 g reactor sludge was exchanged with freshly prepared substrate (HRT of 22 days, similar to that of the large‐scale plant) with a stainless‐steel syringe. After feeding, the conductivity and pH were measured in the samples. For biochemical and molecular biological analyses, 1 mL samples were immediately frozen at −20°C.

For 68 days, both reactors were operated at 55°C; thereafter, the temperature in the reactor (R2_meso_) was lowered to 38°C while R1_thermo_ remained thermophilic (55°C). Prior to the temperature switch, besides monitoring continuous biogas production and quality, the stability of the biogas process in both reactors was biochemically monitored regularly (about four times a week). After the temperature switch in R2_meso_, both reactors were biochemically monitored on Day 68, 70, 71, 72, 76, 77, 78, 84, 98, 100, 101, 104, and 107 (*n* = 26). Samples of Day 68, 77, 84, and 104 (*n* = 8) were used for high‐throughput 16S ribosomal RNA (rRNA) amplicon sequencing and digital polymerase chain reaction (dPCR).

### 2.2 Biochemical analyses

The total solids (TS) and VS content of PW were assessed via standard procedures (Schinner et al., 1996). The biogas composition (H_2_, CH_4_, and CO_2_) was measured with a gas chromatography‐thermal conductivity detector (Wagner et al., 2012; Wagner, Markt, et al., 2019). Volatile fatty acids (VFAs) and organic acids (formate, acetate, propionate, i‐butyrate, n‐butyrate, i‐valerate, n‐valerate, and lactate) as well as phenyl acid (phenylacetic acid [PAA], phenylpropionic acid [PPA], and phenylbutyric acid [PBA]) concentrations were assessed via high‐performance liquid chromatography‐UV/VIS at 220 and 270 nm as described previously (Wagner et al., 2017; Wagner, Prem, et al., 2019). The conductivity of the reactor samples was measured with the conductivity meter LF 330/SET (WTW) and a TetraCon® 325 conductivity cell. The pH meter pH 340i/Set (WTW) with a combined Pt‐ring electrode (®Metrohm Inula) was used for measuring the pH of the sludge samples.

### 2.3 High‐throughput amplicon sequencing and read analyses

Sample preparations and DNA extraction took place according to previously published protocols (Prem et al., 2020) except that the pellet washing step was done with 600 µL phosphate buffer (1X) solution. In brief, the DNA was extracted with a Soil Extract II Kit (Macherey‐Nagel) according to the manufacturer's instructions using lysis buffer SL‐1 and 50 µL enhancer solution. DNA quality and quantity were spectrophotometrically checked with a NanoDrop 2000c™ system (RRID: SCR_020309; Thermo Fisher Scientific™), and fluorometrically with the Quant‐iT™ PicoGreen™ dsDNA Assay Kit (Invitrogen™) and an Anthos‐Zenyth Multimode Detector (Wagner et al., 2015), respectively. In 96 well‐plates, the DNA extracts were diluted to 2.5 ng µL^−1^ with PCR‐grade water via a Biomek 4000 Automated Workstation ((RRID: SCR_019618; Beckman Coulter).

The PCR libraries (first and second PCR steps) were prepared, checked, purified, and pooled according to a previously published study (Prem, Schwarzenberger, et al., 2023). The sequencing library contained samples from several studies (in total: 157 samples). The final ready‐to‐load sample pool showed a DNA concentration of 15 ng µL^−1^ and a 260/280 absorbance ratio of 1.62. The pooled sample was sent to Microsynth AG and sequenced on a MiSeq™ System (RRID: SCR_020134; Illumina®) according to the company's protocols. After sequencing, each study was further analyzed separately.

Reads procession (including preparing a contigs file, quality filtering, preclustering, chimera checks, sequence classification via the k‐nearest neighbor algorithm, assigning to operational taxonomic units [OTUs] according to their taxonomy, rarefaction analyses, and subsampling) was done as described previously (Prem et al., 2019) except that mothur version 1.46.1 (RRID: SCR_011947, Schloss et al., 2009) was used. After quality filtering, 237,920 sequences (25,068 unique sequences) remained for classification. Samples were normalized to 22,601 sequences per sample according to rarefaction analyses. Due to an insufficient sequencing depth of sample R1_thermo_ on Day 84, this time point was excluded from further metagenomic analyses.

### 2.4 dPCR

For absolute quantifications of sequence reads, dPCR was performed on a QIAcuity One 5plex System device (Qiagen: RRID: SCR_008539) with a QIAcuity EvaGreen PCR Kit (QIAGEN). The absolute abundance of genes coding the alpha subunit of the methyl coenzyme M reductase (*mcrA*—primer pair mlas‐f/mcrA‐r) for methanogenic archaea (Steinberg & Regan, 2008) and alpha subunit of the iron hydrogenases (*hydA*—primer pair 1290‐f/1538‐r) for fermentative bacteria (Pereyra et al., 2010) were assessed with an 26k 24‐well nanoplate under following dPCR conditions: 95°C 2 min, followed by 40 cycles at 95°C for 20 s and 60°C for 60 s (annealing and elongation combined, *mcrA*), and 72°C for 5 min. For *hydA*, a three‐step PCR was performed at 58°C for 20 s (annealing) and 72°C for 20 s (elongation). The restriction enzyme EcoRI‐HF (New England Biolabs) was added to the PCR solution (0.25 U µL^−1^); after template addition but prior to loading the nanoplate, the samples were incubated at room temperature for 10 min. For taking pictures after cycling, the exposure duration was set at 200 ms with a gain of 3.

### 2.5 Graphical and statistical analyses

The depiction of biogas, VFA, and dPCR data was done with SigmaPlot™ 15 (RRID: SCR_003210; Systat® Software Inc.). Log_10_ transformation was done for dPCR data which are presented with a 95% confidence interval. Nonmetric multidimensional scaling (NMDS) ordination with biochemical data (conductivity, pH, biogas rate, CH_4_ and CO_2_ concentration, concentrations of VFAs and phenyl acids) were done in R 4.2.3 (RRID: SCR_001905) and RStudio® 2023.03.0 (RRID: SCR_000432) with the packages *readxl* (Wickham & Bryan, 2023), *vegan* (Oksanen et al., 2022), *phyloseq* (McMurdie & Holmes, 2013), *dplyr* (Wickham, Francois, et al., 2023), and *extrafont* (Chang, 2023). Biochemical data were *Hellinger* transformed (Legendre & Gallagher, 2001) prior ordination took place with the *Bray‐Curtis* distance. The heat tree and the heatmap, showing the most abundant taxa (>25 and >200 reads, respectively) over all samples, were also done in R and RStudio® with the packages *tidyverse* (Wickham et al., 2019), *dplyr* and *metacoder* (Foster et al., 2017) for the heat tree, and *readxl*, *pheatmap* (Kolde, 2019), and *extrafont* for the heatmap. Hierarchical clustering was used for defining clusters (heatmap).

## 3 RESULTS

### 3.1 Biochemical data

**Figure 1:**
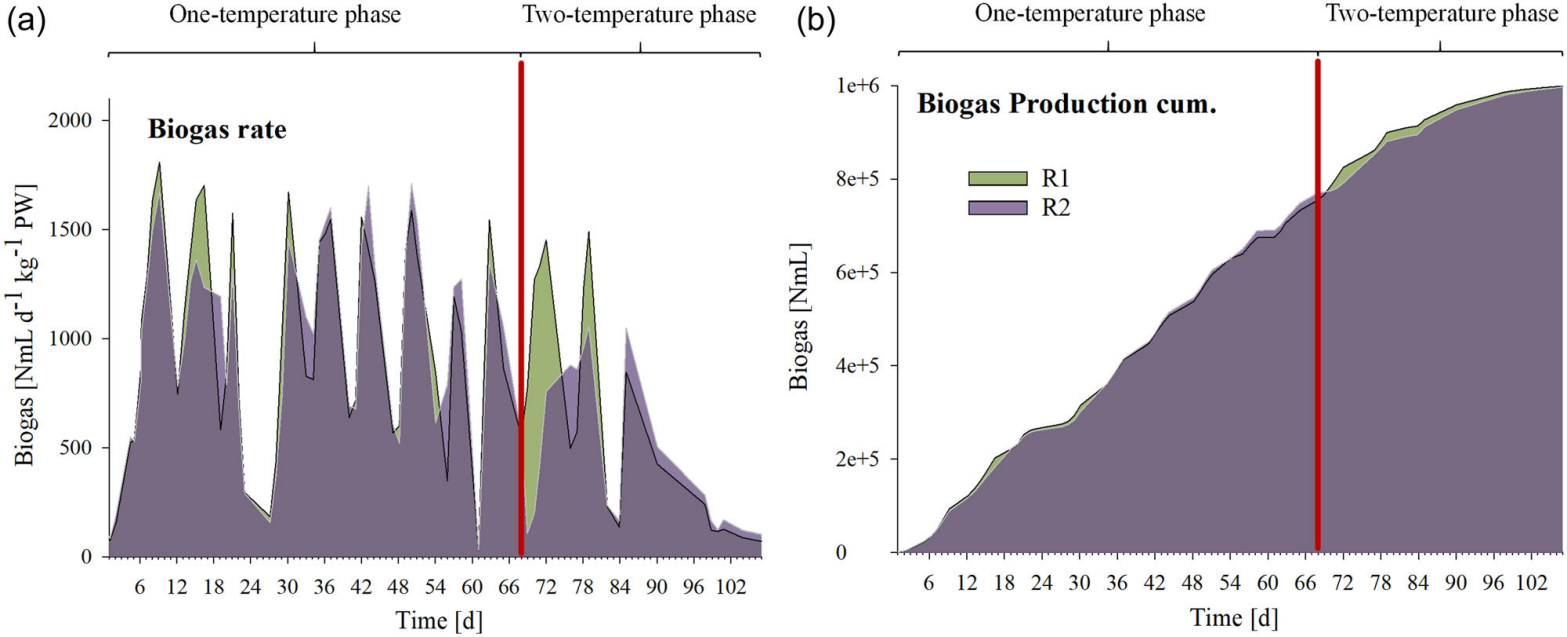
Biogas rates (a) and cumulative biogas production (b) of reactor 1 (R1, green) and reactor 2 (R2, violet) throughout the incubation. On Day 68, the temperature in R2 was switched from 55°C to 38°C (two‐temperature phase → R1_thermo_, R2_meso_). The temperature switch is marked with a red line.

For a list of general biochemical data (biogas rate, CH_4_ and CO_2_ concentrations, conductivity, and pH), and for VFA and phenyl acid concentrations, please refer to Tables 1 and 2, respectively. TS and VS content of undiluted PW (fresh weight, FW) were 19.0 ± 3.45% and 12.5 ± 0.86%, respectively. Methane concentrations ranged from 64.2% to 73.1% and from 63.9% to 77.1% in R1_thermo_ and R2_meso_, respectively. From Day 7 to 65 (stable methane production prior the temperature switch), biogas rates of up to 1.8 (R1) and 1.7 (R2) NL day^−1^ kg^−1^ reactor sludge were observed (Figure 1a) which correspond to 28.9 (R1) and 27.4 NL day^−1^ kg^−1^ volatile solids (VS). After the temperature switch, biogas rates decreased in R2_meso_ by 86% (Day 69), 84% (Day 70), 68% (Day 71), and 48% (Day 72). Thereafter, biogas rates in R2_meso_ increased again and biogas production was even higher by 77% and 50% in R2_meso_ on Day 76 and 77, respectively. The same dynamics were observed when focusing only on methane production (reduction by 85%, 82% 65%, and 51% on Days 69–72, respectively, and an increase of 89% and 58% on Days 76 and 77, respectively). From Day 84 on, R2_meso_ showed a biogas and methane production that was 29 ± 13% higher than in R1_thermo_ (Figure 1a). Cumulatively, R1 produced 1003 and R2 1002 NL biogas per reactor in 111 days (Figure 1b) and 270 and 254 NL in 36 days (after the temperature switch), respectively.

**Table 1:** Biogas production, CH_4_ and CO_2_ concentrations, conductivity, and pH of samples of reactor 1 (R1, gray) and reactor 2 (R2, clear) throughout the incubation.

Time (d)	Biogas R1 (NmL day^−1^ kg^−1^)	Biogas R2 (NmL day^−1^ kg^−1^)	CH_4_ R1 (%)	CH_4_ R2 (%)	CO_2_ R1 (%)	CO_2_ R2 (%)	Conductivity R1 (mS cm^−1^)	Conductivity R2 (mS cm^−1^)	pH R1	pH R2
1.2	73.7	82.3	39.2	40.2	30.9	34.4	18.2	18.4	7.91	7.11
2.1	156	195	47.7	50.2	30.6	29.5	18.1	18.7	7.90	7.80
5.1	534	529	50.5	59.0	27.7	29.5	18.9	19.3	7.85	7.90
6.2	855	798	59.6	63.6	25.9	26.3	18.5	18.7	7.67	7.76
8.0	1630	1496	68.7	67.8	23.6	24.2	18.2	18.4	8.01	8.04
9.2	1808	1675	70.3	74.7	23.0	24.0	17.7	17.9	8.00	8.07
13.3	1127	988	65.3	70.7	25.1	28.1	17.8	18.0	7.91	7.87
14.2	1399	1265	68.3	67.7	25.0	25.1	17.8	18.0	7.96	7.95
15.1	1636	1363	68.3	67.8	26.5	26.8	17.6	18.0	8.01	7.98
16.4	1702	1236	68.2	71.8	26.1	27.6	17.8	18.0	8.01	8.03
19.1	580	1193	62.7	73.4	25.5	24.4	17.4	17.7	8.03	8.12
20.1	839	788	65.4	70.7	26.8	27.1	17.8	17.5	7.92	7.98
23.0	295	292	67.4	69.2	27.3	27.9	17.3	17.0	8.00	8.01
27.1	183	156	60.8	63.8	29.2	23.8	17.1	17.3	7.82	7.88
34.2	811	1021	65.3	70.0	27.7	29.9	19.4	19.2	8.03	7.99
37.0	1546	1600	67.4	71.4	26.0	26.6	19.2	19.4	8.19	8.21
41.1	718	677	63.7	67.5	27.2	28.7	19.1	19.6	7.94	7.90
44.2	1260	1326	68.5	71.6	30.3	26.5	19.4	19.6	7.97	7.94
48.2	599	519	62.2	67.0	28.0	28.2	19.6	19.8	7.89	7.88
51.1	1376	1537	66.2	68.2	28.3	25.2	19.5	19.7	8.00	7.99
62.8	1544	1354	67.8	67.6	28.4	29.4	26.9	28.3	8.01	7.92
68.0	551	558	67.7	68.8	25.3	27.2	28.3	29.0	7.90	7.89
70.1	1272	200	67.1	77.1	24.7	20.9	27.2	25.0	7.92	7.86
71.0	1333	429	68.1	76.2	27.8	23.6	26.3	24.5	8.27	8.02
72.0	1451	758	73.1	70.4	25.4	27.3	27.6	25.7	7.94	7.58
75.9	497	878	68.2	74.4	27.8	24.3	28.9	25.8	7.91	7.73
77.0	571	859	67.3	72.4	29.6	26.9	28.2	26.1	7.96	7.73
78.1	1232	959	69.2	71.5	28.7	26.2	27.5	25.3	7.94	7.72
83.9	136	166	66.8	69.1	29.9	29.5	27.7	26.2	7.93	7.69
97.8	240	281	67.9	67.6	29.9	29.7	29.8	26.7	7.88	7.68
98.8	121	165	64.3	65.4	29.1	28.9	26.9	24.5	7.99	7.75
99.9	116	123	65.5	66.6	28.5	28.5	28.6	25.8	7.97	7.75
100.8	126	170	65.0	63.9	29.3	30.2	28.1	25.1	7.93	7.69
103.9	87.3	122	66.1	65.7	29.2	29.5	29.8	25.8	7.88	7.64
106.8	70.7	104	64.2	64.1	30.4	30.2	29.5	25.8	7.89	7.67

*Note*: On Day 68, the temperature of R2 was changed to 38°C (R2_meso_).

**Table 2:** VFA (lactate, formate, acetate, propionate, i‐butyrate, n‐butyrate, i‐valerate, n‐valerate) concentrations as well as PAA, PPA, and PBA concentrations of samples of reactor 1 (R1, gray) and reactor 2 (R2, clear) throughout the incubation.

Time (day)	Lact R1	Lact R2	Form R1	Form R2	Acet R1	Acet R2	Prop R1	Prop R2	i‐But R1	i‐But R2	n‐But R1	n‐But R2	i‐Val R1	i‐Val R2	n‐Val R1	n‐Val R2	PAA R1	PAA R2	PPA R1	PPA R2	PBA R1	PBA R2
1.2	0.26	0.26	0.00	0.00	9.00	9.00	9.50	10.3	0.35	0.63	0.54	0.40	2.56	2.42	29.2	28.2	4.95	1.48	0.14	0.50	0.41	0.41
2.1	0.00	0.00	0.00	0.00	20.9	21.5	12.9	12.6	0.27	0.40	0.49	0.10	2.40	2.68	32.4	31.6	5.38	1.83	0.20	0.52	0.57	0.56
5.1	0.13	0.19	0.00	0.00	75.7	74.5	14.3	14.5	0.68	0.85	0.07	0.37	2.19	3.09	32.3	33.8	5.32	2.18	0.20	0.14	0.58	0.68
6.2	0.25	0.28	0.00	0.00	72.9	68.6	18.2	18.4	0.87	1.04	0.19	0.00	3.24	3.71	32.6	29.2	5.79	2.47	0.24	0.17	0.72	0.78
8.0	0.27	0.30	0.00	0.00	46.6	43.0	25.8	25.8	0.83	0.74	0.20	0.20	6.08	6.59	36.1	31.5	6.03	2.71	0.50	0.58	0.97	1.33
9.2	0.00	0.00	0.00	0.00	14.0	13.8	30.7	29.4	0.00	0.00	0.12	0.20	6.42	7.08	34.7	34.7	5.89	2.30	0.67	0.93	0.70	1.48
13.3	0.00	0.00	0.00	0.00	2.47	2.49	33.2	35.8	0.00	0.00	0.24	0.00	3.37	3.23	0.00	0.00	5.26	2.61	1.21	0.29	0.71	1.74
14.2	0.00	0.00	0.00	0.00	2.04	2.59	29.9	38.2	0.00	0.00	0.21	0.15	3.15	3.59	0.00	0.00	5.14	2.88	1.53	1.47	0.85	1.92
15.1	0.00	0.00	0.00	0.00	2.77	2.08	22.7	41.8	0.00	0.00	0.00	0.00	3.17	3.86	0.00	0.00	5.20	3.08	1.64	1.54	0.99	2.07
16.4	0.00	0.00	0.00	0.00	1.90	0.00	5.58	38.9	0.00	0.00	0.00	0.00	3.17	3.59	0.00	0.00	5.06	3.15	1.57	1.41	1.12	2.29
19.1	0.24	0.44	0.00	0.00	0.00	0.00	4.08	4.29	0.00	0.00	0.35	0.40	3.74	3.45	0.00	0.00	4.76	2.90	1.44	1.49	1.07	2.39
20.1	0.26	0.43	0.00	0.00	2.50	1.88	5.21	4.54	0.38	0.00	0.07	0.45	3.81	3.41	0.00	0.00	4.88	3.14	1.19	1.54	0.88	2.44
23.0	0.33	0.41	0.00	0.00	0.00	0.00	4.17	4.00	0.00	0.00	0.33	0.41	3.31	3.05	6.66	0.00	4.58	2.90	0.94	1.36	0.95	2.25
27.1	0.32	0.26	0.00	0.00	0.00	0.00	4.11	3.41	0.00	0.00	0.00	0.00	2.94	2.47	20.3	20.6	3.93	2.50	0.49	0.87	0.00	1.37
34.2	0.31	0.29	0.00	0.00	3.51	3.64	3.36	3.64	0.00	0.00	0.00	0.00	2.75	2.94	19.3	18.0	3.43	4.12	0.41	0.40	0.22	0.21
37.0	0.27	0.32	0.00	0.00	0.98	2.29	3.67	3.36	0.00	0.00	0.00	0.00	3.24	3.02	20.0	21.4	4.65	4.30	0.41	0.53	0.22	0.89
41.1	0.32	0.35	0.00	0.00	1.60	1.69	3.71	3.33	0.00	0.00	0.00	0.00	3.37	3.06	17.2	14.1	4.19	3.45	0.42	0.43	0.12	0.10
44.2	0.32	0.44	0.38	0.00	0.00	0.00	2.27	3.34	0.00	0.00	0.00	0.00	3.41	3.20	16.5	19.3	4.32	3.58	0.41	0.49	0.05	0.00
48.2	0.39	0.40	0.00	0.00	1.29	1.60	3.73	3.59	0.00	0.00	0.00	0.00	2.70	2.69	17.4	14.2	3.78	3.29	0.43	0.45	0.12	0.14
51.1	0.00	0.00	0.16	0.33	2.23	2.63	0.00	0.49	2.32	1.59	0.00	0.00	3.82	1.45	–	–	4.14	4.12	0.50	0.42	0.00	0.03
62.8	0.00	0.00	0.06	0.05	3.72	11.7	1.49	1.28	1.44	1.15	0.00	0.00	3.96	4.01	18.1	20.3	3.30	3.74	0.43	0.46	0.00	0.00
68.0	0.00	0.00	0.14	0.42	1.40	0.00	0.00	0.00	1.89	1.84	0.00	0.00	4.09	4.77	15.4	16.7	2.31	3.35	0.44	0.43	0.00	0.00
70.1	0.00	0.00	0.22	0.26	2.40	2.92	0.23	3.84	1.70	0.15	0.00	0.00	3.90	3.03	15.6	18.3	2.53	3.72	0.43	0.43	0.04	0.16
71.0	0.00	0.00	0.18	0.26	3.35	9.56	0.27	11.4	2.09	0.00	0.00	0.00	3.78	1.34	15.4	18.7	2.69	3.95	0.43	0.45	0.03	0.13
72.0	0.00	0.00	0.00	0.18	0.26	24.1	0.00	11.3	1.53	0.31	0.00	0.68	3.86	1.95	15.9	20.4	2.88	4.19	0.44	0.46	0.00	0.10
75.9	0.00	0.00	0.43	0.00	0.00	0.00	0.00	15.8	1.48	0.62	0.00	0.00	3.90	4.07	14.4	19.9	1.39	3.61	0.46	0.38	0.00	0.00
77.0	0.00	0.00	0.29	0.07	2.29	3.44	1.54	18.3	1.02	0.96	0.00	0.00	3.56	3.88	16.3	19.2	1.32	3.47	0.47	0.60	0.06	0.00
78.1	0.00	0.00	0.23	0.27	7.49	3.40	3.36	20.8	0.00	1.19	0.00	0.00	3.78	4.00	15.4	15.9	1.29	3.46	0.48	0.62	0.19	0.00
83.9	0.00	0.00	0.40	0.44	0.00	0.06	0.94	27.9	1.09	0.00	0.00	0.00	4.13	3.08	14.8	13.2	0.04	3.54	0.45	0.37	0.00	0.79
97.8	0.00	0.00	0.42	0.50	0.00	0.00	0.34	0.00	0.16	1.23	0.00	0.00	1.89	4.10	13.5	5.77	0.00	0.43	0.47	0.42	0.00	0.00
98.8	0.00	0.00	0.44	0.50	0.00	0.00	0.40	0.00	0.00	1.23	0.00	0.00	1.78	0.61	13.4	6.02	0.19	0.00	0.47	0.41	0.00	0.00
99.9	0.00	0.00	0.43	0.53	0.00	0.00	0.31	0.00	0.00	0.53	0.00	0.00	1.72	0.50	13.7	6.01	0.32	0.00	0.45	0.41	0.00	0.01
100.8	0.00	0.00	0.48	0.00	0.00	0.00	0.44	0.00	0.00	5.38	0.00	0.00	1.61	0.00	13.5	1.00	0.41	0.05	0.45	0.00	0.00	0.00
103.9	0.00	0.00	0.45	0.58	0.00	0.30	0.70	0.09	0.00	0.34	0.00	0.21	1.22	0.00	13.2	5.89	0.18	0.00	0.42	0.39	0.00	0.00
106.8	0.00	0.00	0.41	0.57	0.00	0.00	2.62	0.23	0.00	0.68	0.00	0.18	0.38	0.00	13.5	5.12	0.00	0.02	0.42	0.38	0.00	0.00

*Note*: Concentrations are shown in mM.

Abbreviations: PAA, phenylacetic acid; PBA, phenylbutyric acid; PPA, phenylpropionic acid; VFA, volatile fatty acid.

**Figure 2:**
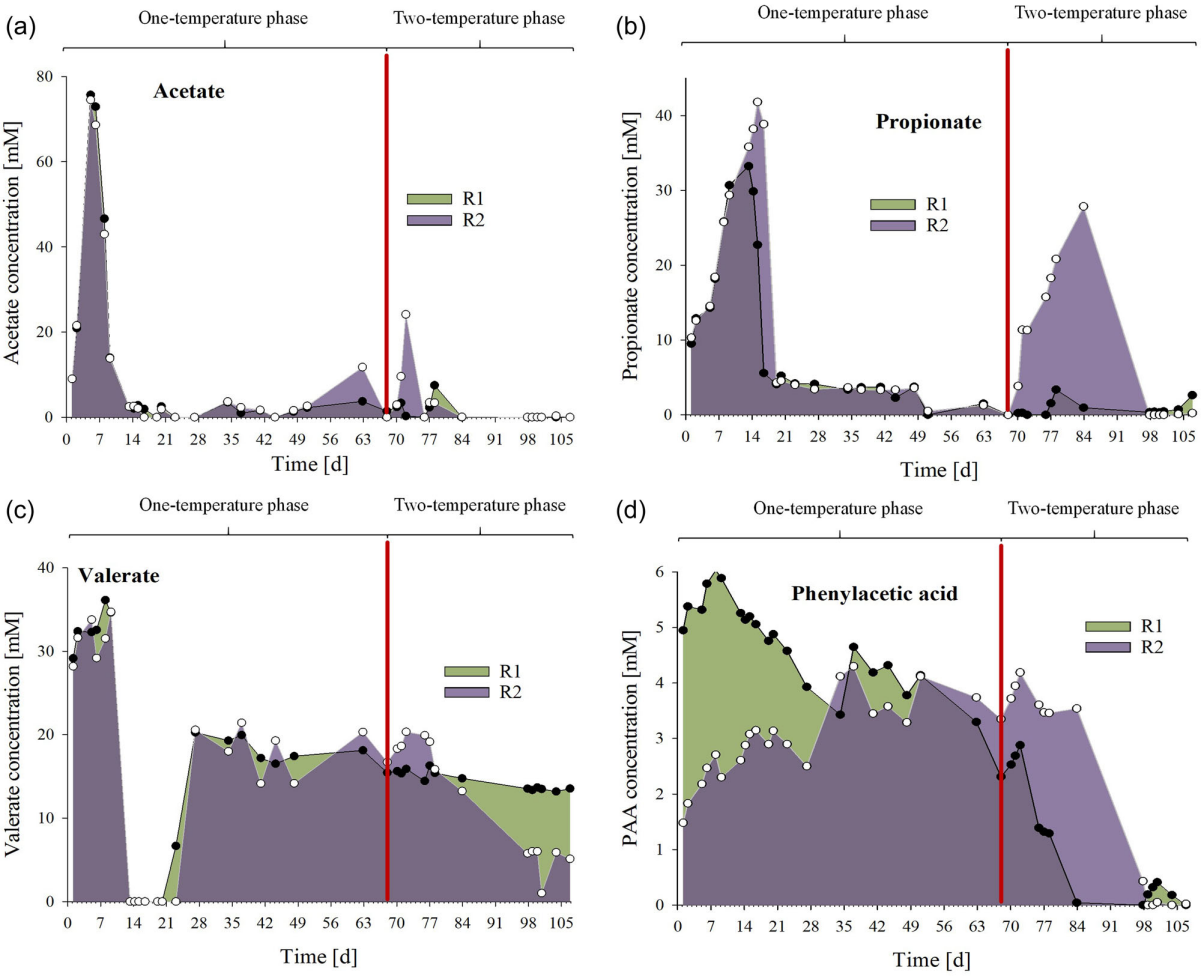
Acetate (a), propionate (b), valerate (c), and phenylacetic acid (d) concentrations during the incubation in reactor 1 (R1, green, filled circle) and reactor 2 (R2, violet, empty circle). On Day 68, the temperature in R2 was switched from 55°C to 38°C (two‐temperature phase → R1_thermo_, R2_meso_). The temperature switch is marked with a red line.

**Figure 3:**
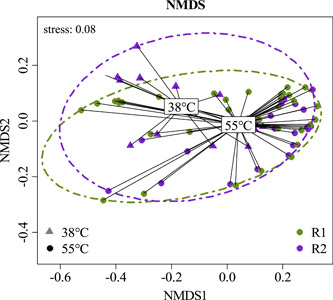
Nonmetric multidimensional scaling (NMDS) analysis of all biochemical data of reactor 1 (R1, green) and reactor 2 (R2, violet) incubated at 55°C (filled circle, all R1 samples as well as R2 samples prior to Day 68) and 38°C (filled triangle, R2_meso_ samples after Day 68). Dashed circles enclose all points of the respective reactor.

The pH was slightly alkalic in both reactors and ranged from pH 7.67 to 8.19 in R1 and from 7.11 to 8.21 in R2 prior temperature switch (Table 1). After changing the temperature in R2_meso_, pH slightly decreased, and values ranged from pH 7.58 to 8.02 (R1_thermo_: pH 7.88– 8.27). The conductivity was also lower in R2_meso_ samples after the temperature switch and ranged from 24.5 to 26.7 mS cm^−1^ (R1_thermo_: 26.3–29.8 mS cm^−1^, Table 1). Acetate and propionate concentrations were high (>12 mM) in both reactors at the beginning of the experiment (up to 75.7 and 74.5 mM acetate, and 33.2 and 41.8 mM propionate in R1 and in R2, respectively) and in R2_meso_ after the temperature switch (up to 24.1 mM acetate on Day 72 and 27.9 mM propionate on Day 84, Table 2, Figure 2). i‐Butyrate concentrations fluctuated in both reactors at low concentrations over the course of the experiment, whereby up to 5.38 mM were temporarily observed in R2_meso_ on Day 101. Valerate concentrations ranged from 14.1 to 21.4 mM in both reactors from Day 27 to 68 (prior to the temperature switch) and decreased in R2_meso_ after changing the temperature (Figure 2c). Phenyl acid concentrations—which can indicate stress during AD (Wagner, Prem, et al., 2019)—were ≤6 mM throughout the incubation; however, PAA concentrations were higher than those of PPA and PBA (Table 2). PAA concentrations decreased in both reactors at the end of the incubation, whereby the decrease in R2_meso_ was time‐delayed (Figure 2d). NMDS ordination with all biochemical data is shown in Figure 3.

### 3.2 16S rRNA amplicon sequencing and dPCR analyses

**Figure 4:**
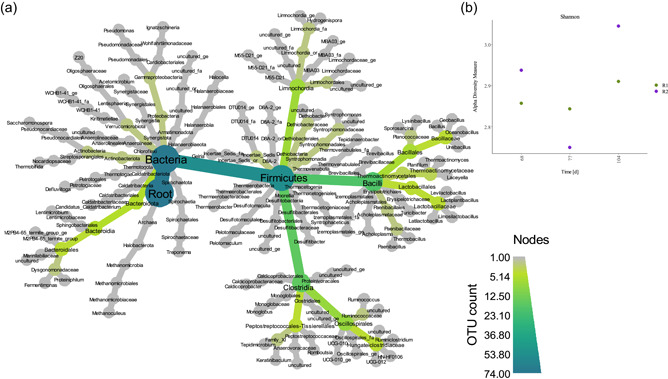
Heat tree (a) showing the microbial community composition of both reactors on Day 68, 77, and 104. Shannon diversity index (b) of R1_thermo_ (green) and R2_meso_ (violet) after the temperature change.

**Figure 5:**
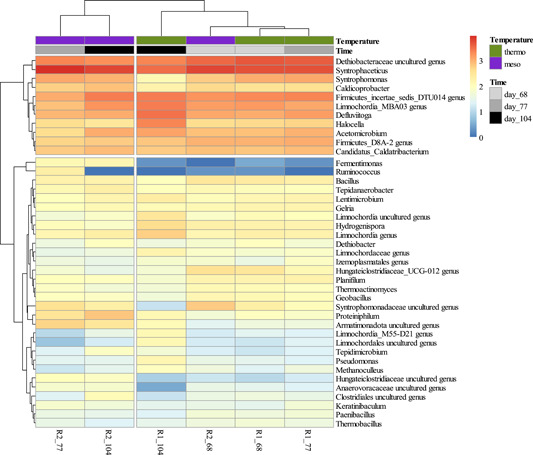
Heatmap of sequence abundance. Microbial taxa with an abundance of >200 sequences per operational taxonomic unit were included in the graph. Read counts were log_10_(*x* + 1) transformed.

After rarefaction analyses, 624 OTUs remained for metagenomic analyses. The most abundant classes were *Bacilli*, *Limnochordia*, *Dethiobacteria*, and *Clostridia*, all belonging the phylum *Firmicutes*, as well as the phylum *Halobacterota*. The alpha diversity measure (Shannon) was lowest in R2_meso_ after 9 days and highest in R2_meso_ after 36 days of mesophilic AD (Figure 4). The core microbiome of all sequencing samples comprised of *Dethiobacteraceae* uncultured, genus *Syntrophaceticus* spp., *Firmicutes*_DTU014 genus, *Limnochordia*_MBA03 genus, *Defluviitoga* spp., *Halocella* spp., *Firmicutes*_D8A‐2 genus, Candidatus *Caldatribacterium* and *Acetomicrobium* spp. The most abundant methanogen was *Methanoculleus* spp. Over all samples, the most abundant genera were *Syntrophaceticus* spp., *Dethiobacteraceae* uncultured genus, *Firmicutes* incertae sedis DTU014 genus, *Limnochordia* MBA03 genus and *Defluviitoga* spp. The abundance of genera like *Fermentimonas* and in part also *Ruminococcus* increased in mesophilic samples over time, whereas abundances were very low in all other samples (Figure 5).

**Figure 6:**
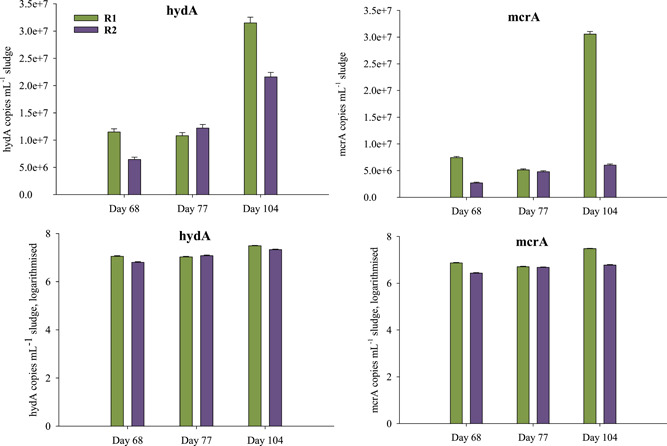
Absolute quantification of *hydA* (left) and *mcrA* (right) sequences mL^−1^ reactor sludge of R1_thermo_ (green) and R2_meso_ (violet) on Day 68, 77, and 104. Top row depicts real copy numbers and the bottom row present log_10_(*x*) data. Whiskers show the respective 95% confidence interval.

Thirty‐six days after the temperature switch, *hydA* copy numbers were 3.15 × 10^7^ (±1.04 × 10^6^) and 2.16 × 10^7^ (±7.78 × 10^5^) mL^−1^ reactor sludge, and *mcrA* copy numbers were 3.06 × 10^7^ (±4.59 × 10^5^) and 6.11 × 10^6^ (±1.41 × 10^5^) mL^−1^ reactor sludge in R1_thermo_ and R2_meso_, respectively (Figure 6).

## 4 DISCUSSION

The aim of this study was to assess the methane production potential of already digested PW from a large‐scale digestion plant and whether another AD process would be useful for more efficient exploitation of PW when substrate loads are high during the touristic high season (winter season 2017/2018). A mesophilic approach was included because biogas production potentials of PW are usually much lower than those of the respective substrate prior to AD, and thermophilic reactors require approximately twice as much energy input than mesophilic ones (Alrowais et al., 2023). In this regard, less energy input for heating and a better exploitation of the residual heat of the incoming PW (in future applications) were thought to be beneficial for an economic, anaerobic posttreatment process. The decrease in temperature from 55°C to 38°C without an adaption period was considered suitable as a previous study showed that a multistep temperature change took longer to reach a stable performance than a one‐step approach. However, the authors of that study increased the temperature which might have had different effects on AD and its microbiome (Boušková et al., 2005). After the temperature switch, both reactors were operated for 36 days. This time frame was considered sufficient for this study, as the HRT at the biogas site is 25−30 days (Illmer & Gstraunthaler, 2009; Weinseisen, 2021).

Acetate, propionate, and valerate concentrations were relatively high at the beginning of the experiment (Figure 2, Table 2) but decreased over time which showed that PW still contained considerable loads of degradable, organic material. This is in correspondence with the high VS content (12.5 ± 0.86% of FW, 65.7 ± 4.51% of TS). The sudden switch to mesophilic AD led to physiological impairments (Figures 1 and 2) in R2_meso_ in the first days: PAA, acetate, propionate, and butyrate concentrations (Figure 2) increased immediately in R2_meso_ thus confirmed their importance as early indicators for process instability during AD (Alavi‐Borazjani et al., 2020; Bonk et al., 2018; Prem, Duschl, et al., 2023; Wagner, Prem, et al., 2019). Besides VFA analyses, monitoring phenyl acid concentrations during AD of complex organic material can give information on the stability of the AD process (Wagner, Prem, et al., 2019). In this study, the detection of PAA is plausible, as PW derived from an OFMSW biogas plant digesting high loads of lignocellulose which renownedly comprises aromatic compounds (Wagner et al., 2014).

Analogously, biogas rates decreased immediately (Figure 1a) after the temperature switch: On Day 69, 70, and 71, R2_meso_ showed biogas production rates reduced by 86%, 84%, and 68% compared with R1_thermo_, respectively. Previous studies concluded that archaeal communities are more sensitive to temperature changes than bacterial ones (Chapleur et al., 2016). This is in accordance with the results of this study, as methanogenesis (decreased biogas rates) was temporarily restricted, and (intermediate) substrates could not be processed as efficiently as prior to the temperature change (VFA accumulations) (Figure 2). Moreover, absolute quantification of *hydA* and *mcrA* sequences further showed that fermentative bacteria (containing the *hydA* sequence) were less impaired by the temperature change than methanogens (containing the *mcrA* sequence) even though differences were generally small between R1_thermo_ and R2_meso_ (see logarithmized data, Figure 6).

Despite these early impairments, low PAA and VFA concentrations (Figure 2) showed stable AD conditions in both reactors at the end of incubation. Concurrently, the methanogenic process recovered, and biogas production rates were even higher in R2_meso_ on some days after the temperature switch (Figure 1). NMDS showed that biochemical data throughout the incubation mainly overlapped (Figure 3). Therefore, despite the short‐term restrictions, an overall difference in biogas production was not observed between R1 and R2 (0.04% reduced biogas yield in the latter). Absolute quantification of the alpha subunit of methyl coenzyme M reductase (*mcrA*) further supported biochemical results and indicated a recovery of methanogenesis in R2_meso_. The higher *hydA* and *mcrA* copy numbers in R1_thermo_ (Figure 6) might be explained by the generally higher cell density and enzyme activity during thermophilic AD (Zhang et al., 2022) as also visible in the Supporting Information S2: Video. On the genus level, a change in the composition of methanogens could not be observed at 38°C, whereby the abundance of *Methanoculleus* spp. slightly decreased in R2_meso_ during the incubation (Figure 5). In this case, 16S rRNA amplicon sequencing did not allow a closer look at *Methanoculleus* species thus it is not clear whether there was a switch from a thermophilic to a mesophilic species or the same species could tolerate lower temperatures (e.g., *M. thermophilus* shows minimum growth below 37°C [Rivard & Smith, 1982]). The second most prevalent methanogen was *Methanomassiliicoccus* spp. but abundance was very low.

During thermophilic AD accumulated substances like valerate (Figure 2c) were exploited more efficiently in the mesophilic digester, which might explain the compensation in biogas production (Figure 1) after switching the temperature to 38° C. Propionate and acetate are the products during β‐oxidation of n‐valerate, and many microorganisms are capable to degrade both, n‐butyrate and n‐valerate (Batstone et al., 2003). Indeed, the increased degradation of n‐valerate after the temperature switch could have contributed to the short‐term increase in propionate and acetate concentrations in R2_meso_ (Figure 2). Thermodynamically, both valerate and propionate are more challenging to degrade during thermo‐ than mesophilic AD (Li et al., 2020). One additional factor for slow valerate degradation during thermophilic AD can be the generally higher propionate levels at those temperatures (Speece et al., 2006) which make the turnover of valerate less feasible. However, in this study, propionate concentrations were even lower in R1_thermo_ than in R2_meso_.

The change in the microbial community might better describe the decrease in valerate concentrations in the mesophilic reactor: Generally, the microbial diversity increased during mesophilic AD (Figure 4) which could have led to a wider spectrum of physiological capabilities. Moreover, the *Firmicutes*:*Bacteroidota* ratio decreased in R2_meso_ with time and was 39 in R1_thermo_ and 14 in R2_meso_ at the end of incubation. A lower ratio is a common characteristic in mesophilic reactors (e.g., Zhang et al., 2019). The abundance of *Fermentimonas* spp., which belongs to the phylum *Bacteriodota*, was increased in R2_meso_ on Day 77 and 104, whereas its contribution was low in all thermophilic samples and in R2_meso_ on Day 68. *Fermentimonas* spp., detected in the human gut (Beye et al., 2018), a lab‐scale mesophilic biogas reactor (Hahnke et al., 2016) and in compost (Duan et al., 2022; Qiu et al., 2022), is a facultative anaerobic acetate producer and degrades carbohydrates as well as complex proteins; moreover, it is closely related to *Proteiniphilum* spp. (Beye et al., 2018; Hahnke et al., 2016). Interestingly, the latter was also more abundant in R2_meso_ than in R1_thermo_ at the end of the experiment (Figure 5). Generally, many representatives of *Bacteriodota* are capable of amino acid degradation (Kuroda et al., 2021), and valerate can be a typical intermediate during these reactions (Kanehisa, 2000). These correlations remain to be verified in further studies. The only described species for *Fermentimonas* spp. so far is *F. caenicola*, which has a temperature optimum at 37−40°C and still grows at 45°C. At this stage, it is not clear whether this organism might survive even higher temperatures (>45°C), or whether the detected one is another, more thermotolerant species.

The main objective of this study was to evaluate meso‐ and thermophilic posttreatment of PW coming from a thermophilic digester. Both temperature regimes showed great potential to posttreat thermophilic PW under anaerobic conditions. In this specific case, physicochemical parameters indicated process impairments in the first days of mesophilic AD. However, these losses in biogas were compensated over time, and mesophilic posttreatment of thermophilic PW reached a similar biogas yield to the thermophilic one after 36 days. We hypothesized that the increased microbial diversity thus the broader spectrum of physiological capabilities and the lower *Firmicutes*:*Bacteriodota* ratio might have enabled the degradation of otherwise recalcitrant substrates, such as valerate. Furthermore, we could show that thermophilic PW can still have enough substrate left for AD—at least at this site in the touristic high season. In previous studies, the liquid fraction was separated from the solid MSW prior to AD and was digested in mesophilic, semicontinuous reactors in the lab. This previously undigested PW reached, at a similar HRT (20 days), a biogas production rate of 696 L biogas kg^−1^ VS day^−1^ (Nayono, Winter, et al., 2010). Here, already digested PW still showed biogas production of up to 28.9 and 27.4 NL biogas kg^−1^ VS day^−1^ in the thermo‐ and mesophilic reactor, respectively, which is about 4% of biogas produced out of undigested PW (Nayono, Winter, et al., 2010).

To our best of knowledge, this was the first study to anaerobically posttreat thermophilic PW at meso‐ and thermophilic temperatures and to add depth by combining biochemical as well as molecular biological parameters. Due to the considerable biogas production potential of PW of this study, biogas potential measurements in touristic regions in Austria throughout the year would be interesting in terms of substrate amount and quality (Illmer & Gstraunthaler, 2009). A (mesophilic) posttreatment after thermophilic AD might be interesting for biogas plant operators not only to yield additional biogas but also to keep undesirable gas emissions at a minimum during storage and field applications. Specific site characteristics like infrastructure or all‐year substrate load have to be assessed individually for each biogas plant. One possible issue would be the availability of additional AD facilities at the site or the profitability of building additional biogas reactors (i) for posttreating PW or to (ii) reduce substrate loads of OFMSW and instead feed another biogas reactor with excess substrates. Moreover, specifications of the respective region must be considered: A mesophilic posttreatment of thermophilic PW might have some advantages as discussed above but might not be congruent with hygiene regulations. In this context, a comprehensive sampling scheme in collaboration with several biogas plant operators is currently planned to finally obtain a comprehensive picture that would be helpful to produce biogas more efficiently from organic (waste) substrates.

## AUTHOR CONTRIBUTIONS

Eva Maria Prem, Rudolf Markt, and Andreas Otto Wagner designed the experiment, set up the reactors, did the sampling, and conducted biochemical analyses. Eva Maria Prem processed and statistically analyzed biochemical data. Eva Maria Prem prepared and checked the library for amplicon sequencing, processed raw sequences, and statistically analyzed sequencing data. Eva Maria Prem and Mathias Wunderer wrote the manuscript draft. Andreas Otto Wagner and Eva Maria Prem raised the funding. Andreas Otto Wagner supervised the findings of the study and the manuscript draft. All the authors read and approved the manuscript.

## CONFLICT OF INTEREST STATEMENT

The authors declare no conflict of interest.

## Supporting information

Video showing a close‐up of the thermophilic reactor R1 in fast motion (4x) during the one‐temperature AD phase. Biogas bubbles are clearly visible and indicate a highly active community digesting residual substrate coming from the thermophilic AD plant.

## Data Availability

The raw sequence reads can be found on the homepage of the National Center for Biotechnology Information under the BioProject (RRID:SCR_004801) Number PRJNA877396 (http://www.ncbi.nlm.nih.gov/bioproject/877396).
